# Plantamajoside alleviates acute sepsis-induced organ dysfunction through inhibiting the TRAF6/NF-κB axis

**DOI:** 10.1080/13880209.2023.2215849

**Published:** 2023-06-08

**Authors:** Daili Feng, Ruying Guo, Wei Liao, Jiancheng Li, Song Cao

**Affiliations:** Department of Emergency, Rongchang People’s Hospital, Chongqing, China

**Keywords:** Traditional Chinese medicine, organ damage, apoptosis, systemic inflammation

## Abstract

**Context:**

Plantamajoside (PMS) possesses rich pharmacological characteristics that have been applied to remedy dozens of diseases. However, the understanding of PMS in sepsis remains insufficient.

**Objective:**

Role of PMS in sepsis-regulated organ dysfunction and potential mechanisms were investigated.

**Materials and methods:**

Thirty C57BL/6 male mice were adaptive fed for three days and used to establish acute sepsis model by caecal ligation and perforation (CLP). These experimental mice were divided into Sham, CLP, CLP + 25 mg PMS/kg body weight (PMS/kg), CLP + 50 mg PMS/kg and CLP + 100 mg PMS/kg (*n* = 6). The pathological and apoptotic changes of lung, liver and heart tissues were observed via HE and TUNEL staining. The injury-related factors of lung, liver and heart were detected by corresponding kits. ELISA and qRT-PCR were applied to assess IL-6/TNF-α/IL-1β levels. Apoptosis-related and TRAF6/NF-κB-related proteins were determined using Western blotting.

**Results:**

All doses of PMS enhanced the survival rates in the sepsis-induced mouse model. PMS remitted sepsis-mediated lung, liver and heart injury through prohibiting MPO/BALF (70.4%/85.6%), AST/ALT (74.7%/62.7%) and CK-MB/CK (62.3%/68.9%) levels. Moreover, the apoptosis index (lung 61.9%, liver 50.2%, heart 55.7% reduction) and IL-6/TNF-α/IL-1β levels were suppressed by PMS. Furthermore, PMS lowered TRAF6 and p-NF-κB p65 levels, whereas TRAF6 overexpression reversed the protective influences of PMS in organ injury, apoptosis and inflammation triggered by sepsis.

**Discussion and conclusions:**

PMS suppressed sepsis-induced organ dysfunction by regulating the TRAF6/NF-κB axis, and PMS treatment may be considered as a novel strategy for sepsis-caused damage in future.

## Introduction

Sepsis is a syndrome of uncontrolled systemic inflammation and often arises from infection, which has become a major lethal reason for patients in intensive care units (ICU) (van der Poll et al. 2021). On the basis of an epidemiological survey, there are about 48 million cases of sepsis worldwide (Gomanova and Brazhnikov 2021), and the mortality dynamics of sepsis have become increasingly prominent due to the diversified complications (Torres et al. 2022). Approximately 8.1 million patients died of sepsis with the mortality rate remaining at 25%–30% in 2021 (Fleischmann et al. 2016; Wong et al. 2021). Currently, sepsis remains an unresolved public health problem in many countries of the world. Organ damage is a principal feature of sepsis, which can further develop multiple organ dysfunction syndrome (MODS) (Sygitowicz and Sitkiewicz 2020). The dominating treatment strategies of sepsis rely on ventilator and continuous renal replacement therapy (CRRT) (Romagnoli et al. 2018; Lourbopoulos et al. 2021), but valid drugs for sepsis is still lacking. Therefore, the search for effective drugs for treatment of sepsis-induced organ damage is extremely important.

Recent investigations indicate that the advantages of traditional Chinese medicine (TCM) components with multiple targets, effects and levels give play to a huge effect in curing sepsis (Wen et al. 2021). The protective functions of familiar TCMs, including *Radix isatidis* from the root of *Isatis tinctoria* L. (Brassicaceae) (Ruan et al. 2020) and *Rheum palmatum* L. (Polygonaceae) (Meng et al. 2020), have been certified in sepsis-induced injury by controlling the process of immune, inflammation and apoptosis. An *in vivo* experiment revealed that Bai-hu-tang (TCM formula) enhanced the survival rate and reduced the accumulation of IL-6 and IL-10 in a sepsis rat model (Lin et al. 2013). Plantamajoside (PMS) is the dominant component of *Plantago asiatica* L. (Plantaginaceae), which has abundant biological activities such as anti-inflammation, anti-apoptosis, antitumour, oxidation resistance and damage repair function (Zuo et al. 2021). The protective effect of PMS in H9c2 cells against hypoxia/reoxygenation (H/R)-triggered injury *in vivo* was reported by Zeng et al. (2022), and the AKT/Nrf2/HO-1 and NF-κB pathways were joined in regulating the protective function of PMS in H9c2 cells. Additionally, Shang et al. (2019) found that PMS alleviated isoproterenol-triggered cardiac dysfunction, which was linked with AKT/GSK-3β pathway. The aforementioned conclusions hinted that PMS could ease lung, liver and heart damage by regulating significant pathways, but the roles of PMS in sepsis are still unclear, and whether PMS influences organ damage by regulating the correlated pathway is worth exploring.

Hence, the *in vivo* experiments were carried out by establishing caecal ligation and puncture (CLP) model to explore the influences of PMS in lung, liver and heart tissues, as well as unearthed the anti-inflammatory and anti-apoptosis effects of PMS in sepsis and TNF receptor associated factor 6 (TRAF6)/NF-κB regulated mechanism. The survey might provide a new direction for the clinical search for valid drugs to treat sepsis.

## Materials and methods

### Construction, grouping and administration of animal model

Thirty C57BL/6 male mice (SPF, 8–10 weeks old, 20–22 g) from SLAC Laboratory Animal Co., LTD (Shanghai, China) were applied to the experiments. The CLP model was constructed to mimic the progression of sepsis. All mice were fed with free access for drinking and eating. After three days, experimental mice were anaesthetized by 1% pentobarbital sodium (CAS:4390-16-3, Sigma, St. Louis, MO, USA), and the lower section of the mouse abdominal cavity was opened to expose the caecum. Subsequently, the caecum was ligated with surgical sutures at a distance of 1.5 cm from the distal caecum. The remnant was punctured with a 22-gauge needle. Afterward, the caecum was placed back into the abdominal cavity and the abdomen was sutured. Mice in Sham group was opened abdomen after anaesthesia without caecal ligation and puncture. The animal experiments were approved by the Ethics Committee on Animal Experiments of Rongchang People’s Hospital (2020-RCRMYY-016).

PMS (CAS: 104777-68-6, purity > 98%) was purchased from Rayzbio Technology Co., Ltd. (Shanghai, China). Mice were divided into five groups, as Sham, CLP, CLP + 25 mg PMS/kg body weight (PMS/kg), CLP + 50 mg PMS/kg and CLP + 100 mg PMS/kg (*n* = 6). Experimental mice in PMS group were administrated by intraperitoneal injection. Mice in the Sham and CLP groups were intraperitoneally injected with an equal volume of normal saline. The survival percentages in each group were assessed at seven time points of 0, 4, 8, 12, 16, 20 and 24 h after modelling and treatment.

### Collection of lung, liver and heart tissues

The experimental mice were deeply anaesthetized by intravenous injection of 100 mg/kg phenobarbital sodium (Sigma). Lung, liver and heart tissues from these mice were taken out and cryopreserved, which were applied to histopathological and Western blotting assays. The mouse blood and bronchoalveolar lavage fluid (BALF) were centrifuged, and the supernatant of BALF and serum was collected for the detection of inflammatory factors. All animal experiments abided by the Ethics Committee on Animal Experiments of Rongchang People’s Hospital.

### Establishment of transfection system

The overexpressed transfection system of TRAF6 were constructed by using ad-TRAF6 vectors. The ad-TRAF6 vectors and the blank contrasted vectors (ad-NC) were achieved from GenePharma (Shanghai, China). The transfection process was performed relying on Lipofectamine 3000 (L3000-015, Invitrogen, Carlsbad, CA, USA).

### Haematoxylin and eosin (H&E) staining

Lung, liver and heart tissues fixed with 4% paraformaldehyde (P6148, Sigma) were cut and dehydrated. These tissues were then waxed into wax blocks. The blocks were cut into 5 μm slices by slicer and then dewaxed, rehydrated and stained for 3 min with haematoxylin (G1120, Solarbio, Beijing, China). Following, the lung, liver and heart sections were differentiated with 1% hydrochloric acid alcohol for 20 s and were stained for 1 min with eosin (G1120, Solarbio). After washing, these tissues were dehydrated with ethanol solution of different concentrations (80%, 90%, 95%, 100%) at room temperature. After soaking in xylene for 10 min, the sections were sealed with neutral gum. The pathological alterations were observed and photographed by an optical microscope (DIAPHOT-TMD, Nikon, Osaka, Japan). The scoring standard of inflammatory infiltration was almost invisible marking 0, mild inflammatory infiltration marking 1, moderate inflammatory infiltration marking 3, and severe inflammatory infiltration marking 5.

### Assessment of lung, liver and heart functions

For evaluation of lung, liver and heart functions, the activity assay kits of myeloperoxidase (MPO) (#ab105136, Abcam, Cambridge, MA), aminotransferase (AST) (#ab263882, Abcam), alanine aminotransferase (ALT) (#ab285263, Abcam), creatine phosphokinase isoenzyme (CK-MB) (#ab285231, Abcam), creatine kinase (CK) (#ab155901, Abcam) were applied for examining the correlated indexes. Total protein in BALF was determined *via* using BCA protein kit (#ab207002, Abcam). An automatic biochemical analyser (AU800; Olympus Corporation, Tokyo, Japan) was used for index analysis.

### TUNEL staining

After grouping and PMS management, the experimental lung, liver and heart tissues were fixed with 4% paraformaldehyde (Sigma). After washing with PBS, 50 µL TUNEL solution was used to stain tissues. The implementation process was in the light of One Step TUNEL Apoptosis Assay Kit (C1088, Beyotime, Shanghai, China). The positive TUNEL-labelled cells were observed *via* using a fluorescence microscope (Eclipse E800, Nikon, Tokyo, Japan).

### ELISA

Following centrifugation (500 *g* for 10 min at 4 °C), the serum from experimental samples was collected and stored at −80 °C. The levels of IL-6, TNF-α and IL-1β were examined through utilizing an IL-6 kit (#ab222503, Abcam), TNF-α kit (#ab208348, Abcam) and IL-1β kit (#ab197742, Abcam).

### qRT-PCR

TRIzol reagent (#15596-026, Invitrogen) was adopted to separate total RNA from lung, liver and heart tissues. Reverse Transcription Kit (#218061, Qiagen, Valencia, CA, USA) was utilized to reverse-transcribe RNA into cDNA. Subsequently, the SYBRGreen PCR kit (SR1110, Solarbio) was applied and qPCR amplification was carried out on a Real-time PCR detector (Roche 480, Mannheim, Germany). The mRNA levels of IL-6, TNF-α and IL-1β were examined by 2^−ΔΔC^_t_ method, and β-actin was served as the reference gene. The primer sequences used in the research included: IL-6-FW, *5′*-TGA TGG ATG CTT CCA AAC TG-*3′*, IL-6-RW, *5′*-GAG CAT TGG AAG TTG GGG TA-*3′*; TNF-α-FW, *5′*-ACT GAA CTT CGG GGT GAT TG-*3′*, TNF-α-RW, *5′*-GCT TGG TGG TTT GCT ACG AC-*3′*; IL-1β-FW, *5′*-CAC CTT CTT TTC CTT CAT CTT TG-*3′*, IL-1β-RW, *5′*-GTC GTT GCT TGT CTC TCC TTG TA-*3′*; β-actin-FW, *5′*-TCC TGT GGC ATC CAC GAA ACT-*3′*, β-actin-RW, *5′*-GAA GCA TTT GCG GTG GAC GAT-*3′*.

### Western blotting

Fifty mg lung, liver and heart tissues of mice were accurately weighed and then were fully ground into powders using a mortar and pestle (BKMAM, Changsha, Hunan). Total proteins from these tissues were prepared *via* utilizing RIPA buffer (R0010, Solarbio) with phenylmethanesulphonyl fluoride (#78830, PMSF, Sigma). After centrifugation for 10 min, the supernatant was taken out and BCA protein quantitative assay kit (PC0020, Solarbio) was applied to determine the protein concentrations. The SDS-PAGE gel was prepared, and the 50 μg total protein was added to the sample well using a microsampler. Protein samples were then separated by electrophoresis and transferred to PVDF membranes. After blocking membranes with 5% BSA, the primary antibodies of cleaved caspase 3 (ab214430, 1:5000, 31 kDa, Abcam), TRAF6 (ab40675, 1:5000, 63 kDa, Abcam), p-NF-κB p65 (ab239882, 1:2000, 65 kDa, Abcam), NF-κB p65 (ab32536, 1:1000, 65 kDa, Abcam) and β-actin (ab6276, 1:5000, 42 kDa, Abcam) were incubated with membranes over night at 4 °C. Subsequently, the relevant second antibody (ab6721, 1:2000, 37 kDa, Abcam) was used to incubate the above membranes for 2 h. The protein signals were observed by the enhanced chemiluminescence (ECL) reagents (NEL105001EA, PerkinElmer, Waltham, MA, USA). Image Lab 6.0.1 software (Bio-Rad, Richmond, CA, USA) was used for grey scale analysis of the Western blotting images.

### Statistical analysis

The SPSS 17.0 software (SPSS, Chicago, IL, USA) was used to analyse the data, which was presented as mean ± SD. The survival rates were calculated *via* utilizing Kaplan Meier method and the log-rank statistics. By means of one-way analysis of variance (ANOVA) and the Turkey’s *post hoc* test, the statistical differences in the different groups were computed. *p*** **<** **0.05 was represented as a statistically significant result.

## Results

### PMS extended survival in CLP model

**Figure 1.**
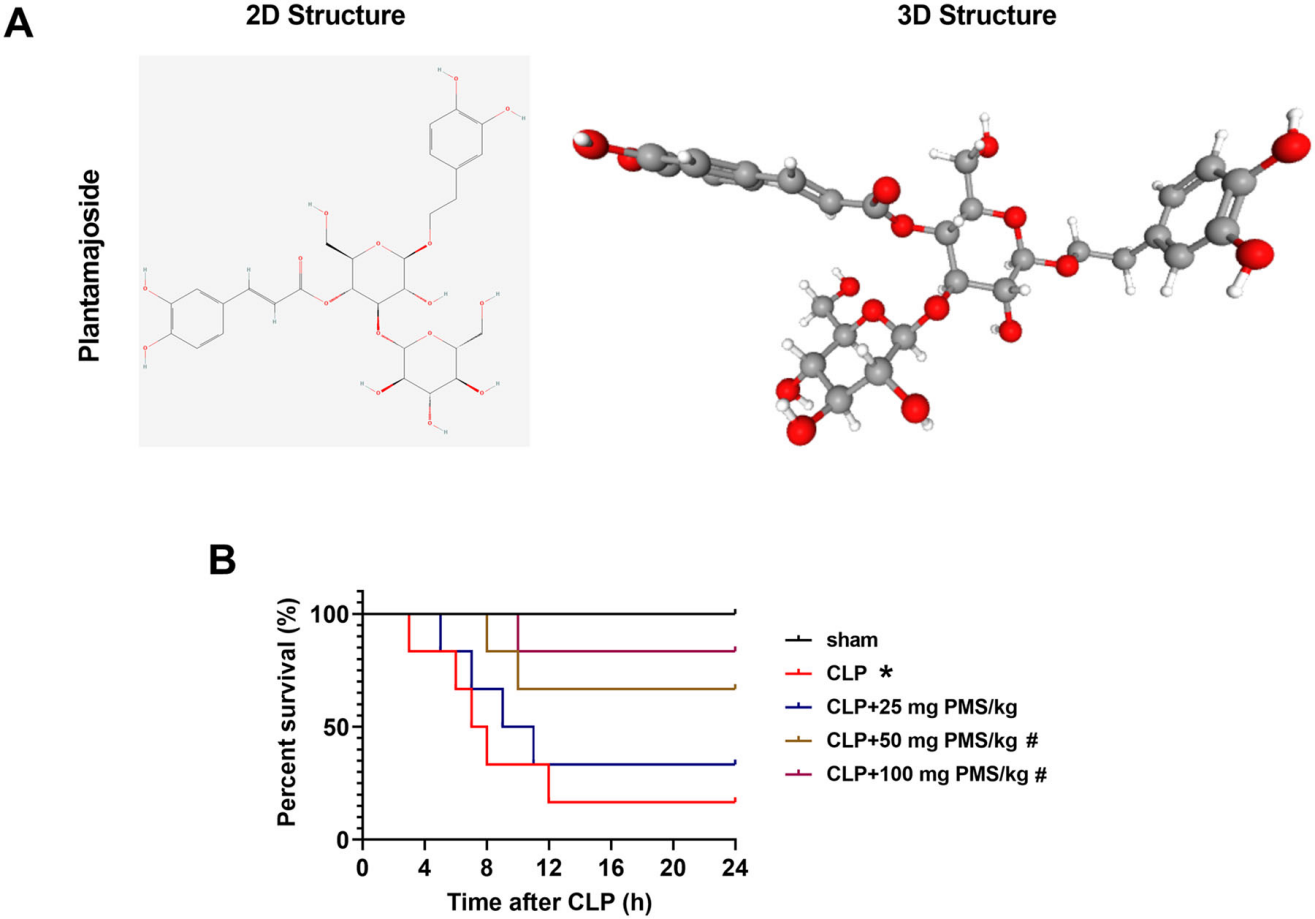
PMS regulated the survival rate of CLP model. (A) The 2D and 3D structure of PMS; (B) The sepsis model *in vivo* was established via CLP method. The experimental mice were then divided into five groups (Sham, CLP, CLP + 25 mg PMS/kg, CLP + 50 mg PMS/kg and CLP + 100 mg PMS/kg). After modelling for 24 h, the survival percentage (%) of mice was evaluated by the Kaplan Meier method. *p < 0.05, *vs.* Sham group; ^#^*p* < 0.05, *vs.* CLP group.

PMS (C_29_H_36_O_16_) possesses high medicinal value and extensive pharmacological action, which has been applied to treat various diseases. The 2D and 3D structures of PMS were shown in Figure 1(A). To research the effect of PMS in sepsis-triggered organ damage, we firstly constructed a CLP model, and the various concentrations (25, 50 and 100 mg PMS/kg) of PMS were used for treatment of CLP mice for 24 h, the survival rates of experimental mice were then investigated. Compared to the Sham group, the survival percentages were significantly reduced in other groups (*p*** **<** **0.05, Figure 1(B)). After intervention with PMS (50 and 100 mg PMS/kg), the survival percentages were obviously increased compared with CLP group (*p*** **<** **0.05, Figure 1(B)). The conclusion indicated that PMS could prolong the survival rates of sepsis-injured mice.

### PMS improved acute sepsis-triggered organ damage

**Figure 2.**
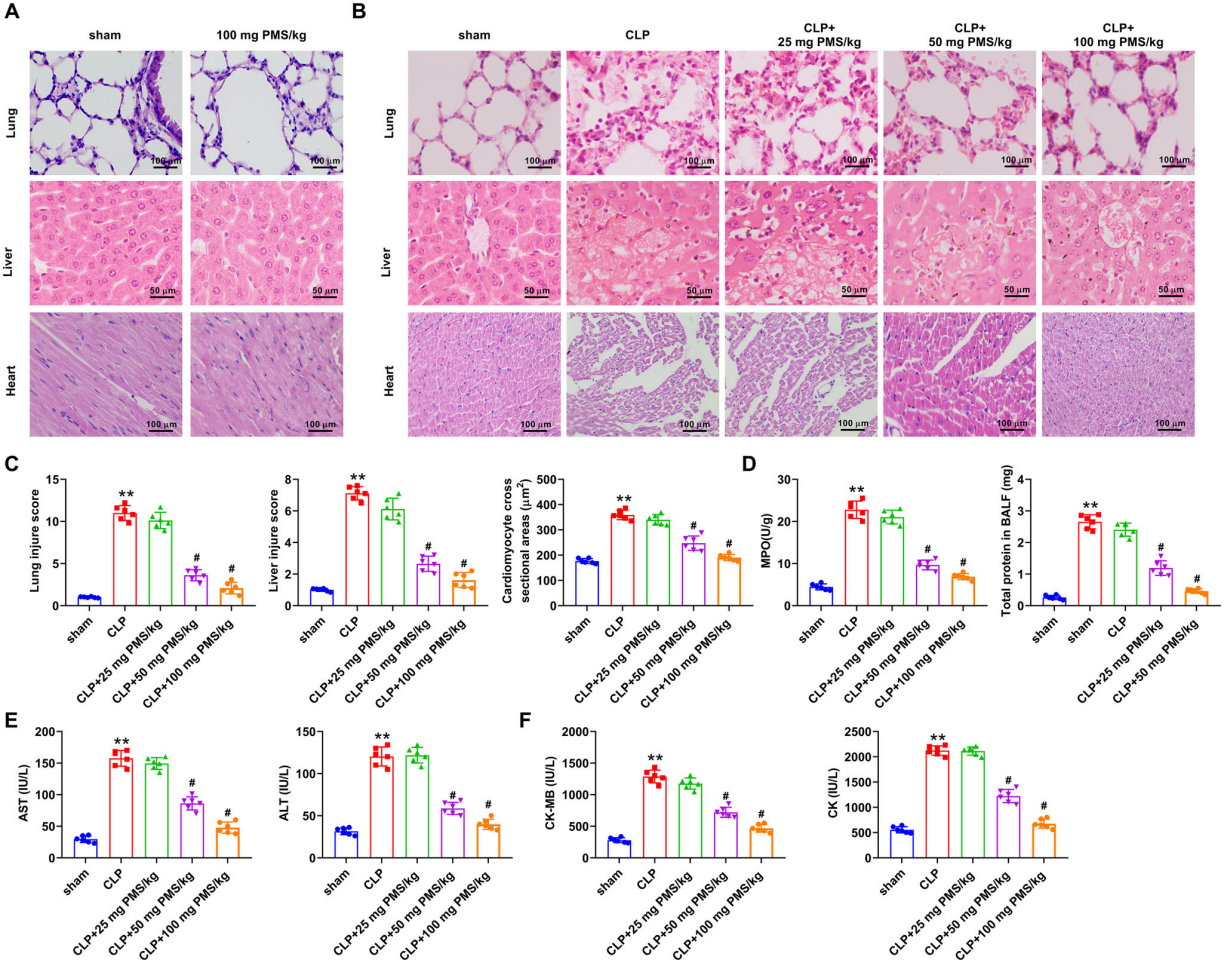
PMS attenuated organ damage in the CLP model. The CLP model was constructed to mimic the occurrence of sepsis, and PMS (25, 50 and 100 mg PMS/kg) was intraperitoneally injected into the experimental mice. The experimental mice were divided into five groups (Sham, CLP, CLP + 25 mg PMS/kg CLP + 50 mg PMS/kg and CLP + 100 mg PMS/kg). (A and B) The damaged condition of lung, liver and heart in these groups were investigated by HE staining, as well as (C) the injury scores of lung, liver and myocardium tissues were assessed (×400 magnification, scale bar: 50 or 100 μm). (D) MPO activity and BALF protein levels in lung tissues were tested via Kit method. (E) The levels of AST and ALT in liver tissues were analysed by the correlated kits. (F) The levels of CK-MB and CK in myocardium tissues were examined by the matched kits. *p < 0.01, *vs.* Sham group; ^#^*p* < 0.05, *vs.* CLP group.

Subsequently, the functions of PMS in sepsis-mediated tissues damage of lung, liver and heart *in vivo* were explored. To exclude the toxic effects, 100 mg PMS/kg was preliminarily utilized to treat experimental mice. Compared to the Sham group, PMS treatment had no significant effect on the lung, liver and heart tissues of experimental mice (Figure 2(A)). In Figure 2(B), HE staining result revealed that the alveolar structure was intact, cells of liver and myocardium was normal and arranged neatly in the Sham group. But, the morphological structures of alveolar, liver and myocardium were clearly damaged in the CLP group. After treatment with PMS, the damaged structures significantly recovered. Additionally, the injury scores of lungs, liver and cardiomyocyte were observably increased in the CLP group compared with that in the Sham group (*p*** **<** **0.001). However, these scores were statistically significantly reduced by the treatment with 50 and 100 mg PMS/kg (*p*** **<** **0.05, Figure 2(C)). Furthermore, the correlated factor levels of lung (MPO and BALF), liver (AST and ALT) and heart (CK-MB and CK) were significantly (*p*** **<** **0.01) enhanced in CLP model, but these were lowered by PMS (50 and 100 mg PMS/kg) treatment (*p*** **<** **0.05, Figure 2(D–F)). The consequences indicated that PMS could alleviate organ damage in the CLP model.

### PMS alleviated acute sepsis-triggered apoptosis

**Figure 3.**
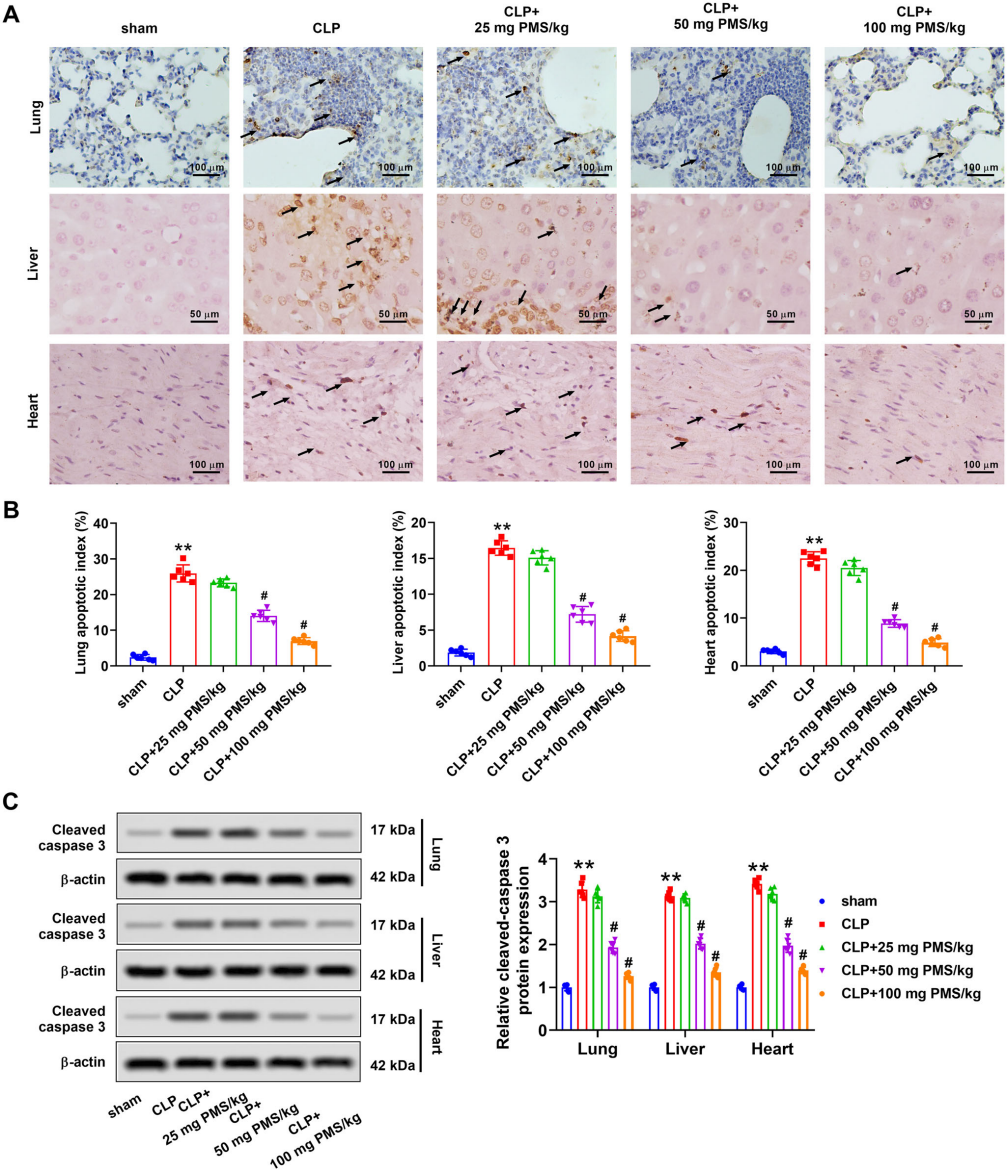
PMS eased lung, liver and heart tissues apoptosis in the CLP model. After modelling and treatment, (A) TUNEL analytical method was applied to evaluate the apoptotic levels of lung, liver and heart tissues (×400 magnification, scale bar: 50 or 100 μm). (B) The apoptotic indexes in lung, liver and heart tissues were computed in different groups. (C) Western blotting assay was used to assess the protein levels of cleaved caspase 3 in lung, liver and heart tissues in above five groups. *p < 0.01, *vs.* Sham group; ^#^*p* < 0.05, *vs.* CLP group.

Functions of PMS in sepsis-triggered apoptosis *in vivo* were next investigated. TUNEL assay uncovered that the number of TUNEL-positive cells from lung, liver and heart tissues prominently increased in the CLP group, relative to that in the Sham group. Observably, the TUNEL-positive cells in PMS-disposed groups were decreased (Figure 3(A)). Similarly, the apoptotic indexes in lung, liver and heart tissues were significantly ascended in CLP group (*p*** **<** **0.01), whereas declined by PMS interference with a concentrated-dependent manner (*p*** **<** **0.01, Figure 3(B)). Western blotting results displayed that cleaved caspase 3 protein levels of lung, liver and heart tissues were significantly upgraded in the CLP model (*p*** **<** **0.01). PMS induction reversed the increased tendency of cleaved caspase 3 protein levels (*p*** **<** **0.05, Figure 3(C)). These results indicated that PMS could improve the apoptosis condition in CLP model.

### PMS relieved acute sepsis-triggered inflammatory response

**Figure 4.**
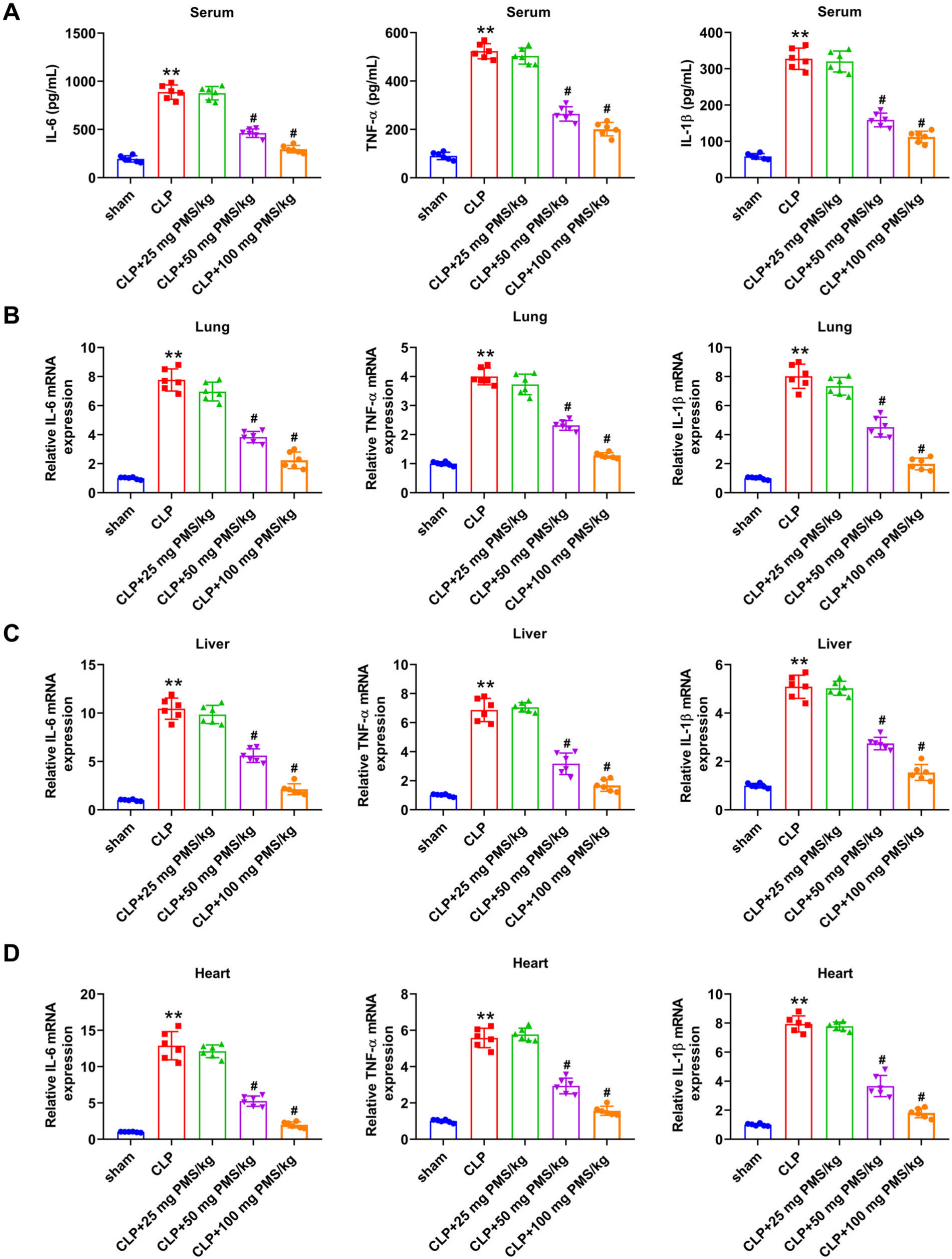
PMS relieved the inflammatory response in the CLP model. After modelling and management, (A) The serum accumulated levels of IL-6, TNF-α and IL-1β were analysed by ELISA. (B–D) The relative mRNA levels of IL-6, TNF-α and IL-1β in lung, liver and heart tissues were studied by RT-qPCR. *p < 0.01, *vs.* Sham group; ^#^*p* < 0.05, *vs.* CLP group.

Excessive activation of inflammatory cells and release of plentiful inflammatory cytokines is a crucial mechanism of the occurrence and development of sepsis. Herein, we investigated the influence of PMS in inflammatory response by determining IL-6, TNF-α and IL-1β expression. ELISA assay results from Figure 4(A) revealed that the concentrations of IL-6, TNF-α and IL-1β in serum were significantly elevated in the CLP group compared with that in Sham group (*p*** **<** **0.01). After disposing with PMS (50 and 100 mg/kg), the accumulation of above inflammatory factors was obviously reduced (*p*** **<** **0.05). In the aspect of mRNA expression from lung, liver and heart tissues, the incremental levels of IL-6, TNF-α and IL-1β presented in the CLP group (*p*** **<** **0.01), but were distinctly reduced in PMS-treated groups (*p*** **<** **0.05, Figure 4(B–D)). These outcomes indicated that PMS could mitigate the inflammatory response in the CLP model.

### PMS improved acute sepsis-triggered organ damage via mediating the TRAF6/NF-κB pathway

**Figure 5.**
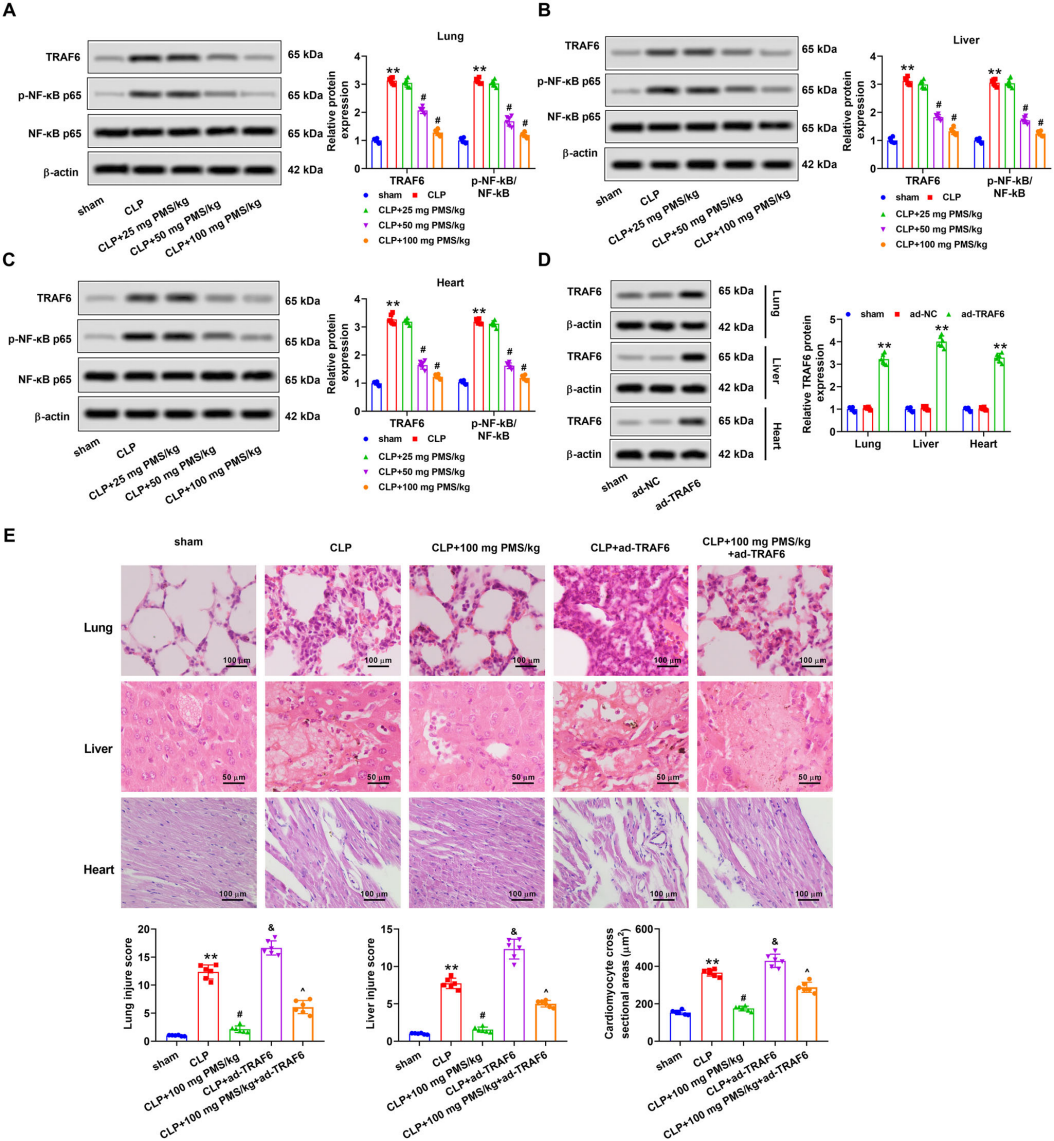
PMS mitigated sepsis-triggered organ damage *via* regulating the TRAF6/NF-κB pathway. After CLP model construction and PMS treatment, (A–C) Protein levels of TRAF6, p-NF-κB p65 and NF-κB in lung, liver and heart tissues were determined via Western blotting. After transfection with ad-TRAF6 and ad-NC, (D) protein levels of TRAF6 in lung, liver and heart tissues were determined via Western blotting. The mice were divided into sham, CLP, CLP + 100 mg PMS/kg, CLP + ad-TRAF6 and CLP + 100 mg PMS/kg + ad-TRAF6 groups. (E) HE staining method was utilized to detect lung, liver and heart tissues injury in different groups of mice, and the injury scores were counted in lung, liver and myocardium (×400 magnification, scale bar: 50 or 100 μm). *p < 0.01, *vs.* Sham or ad-NC group; ^#,&^*p* < 0.05, *vs.* CLP group; ^^^*p* < 0.05, *vs.* CLP + ad-TRAF6 group.

For disclosing the regulatory mechanism of PMS in sepsis-induced organ damage, functions of the TRAF6/NF-κB pathway in this process were studied. Western blotting analytical results displayed that protein levels of TRAF6 and p-NF-κB p65 in lung, liver and heart tissues of the CLP group clearly increased (*p*** **<** **0.01 vs. Sham group). Nevertheless, PMS management dramatically lowered TRAF6 and p-NF-κB p65 protein levels in the lung, liver and heart tissues (*p*** **<** **0.05 vs. CLP group, Figure 5(A–C)). Next, ad-TRAF6 vector was constructed to overexpress TRAF6 expression, and Western blotting results confirmed that the transfected ad-TRAF6 in lung, liver and heart tissues was successful (*p*** **<** **0.01 vs. ad-NC group, Figure 5(D)). HE staining consequence indicated that the damaged degree of lung, liver and heart tissues in CLP + ad-TRAF6 group increased clearly (*p*** **<** **0.05 vs. CLP group). In CLP + PMS (100 mg PMS/kg) + ad-TRAF6 group, the above phenomena were overtly reversed (*p*** **<** **0.05 vs. CLP + ad-TRAF6 group, Figure 5(E)). These results uncovered that the TRAF6/NF-κB pathway participated in regulating the function of PMS in sepsis-triggered organ damage.

### PMS improved acute sepsis-triggered apoptosis and inflammation via regulating the TRAF6/NF-κB pathway

**Figure 6.**
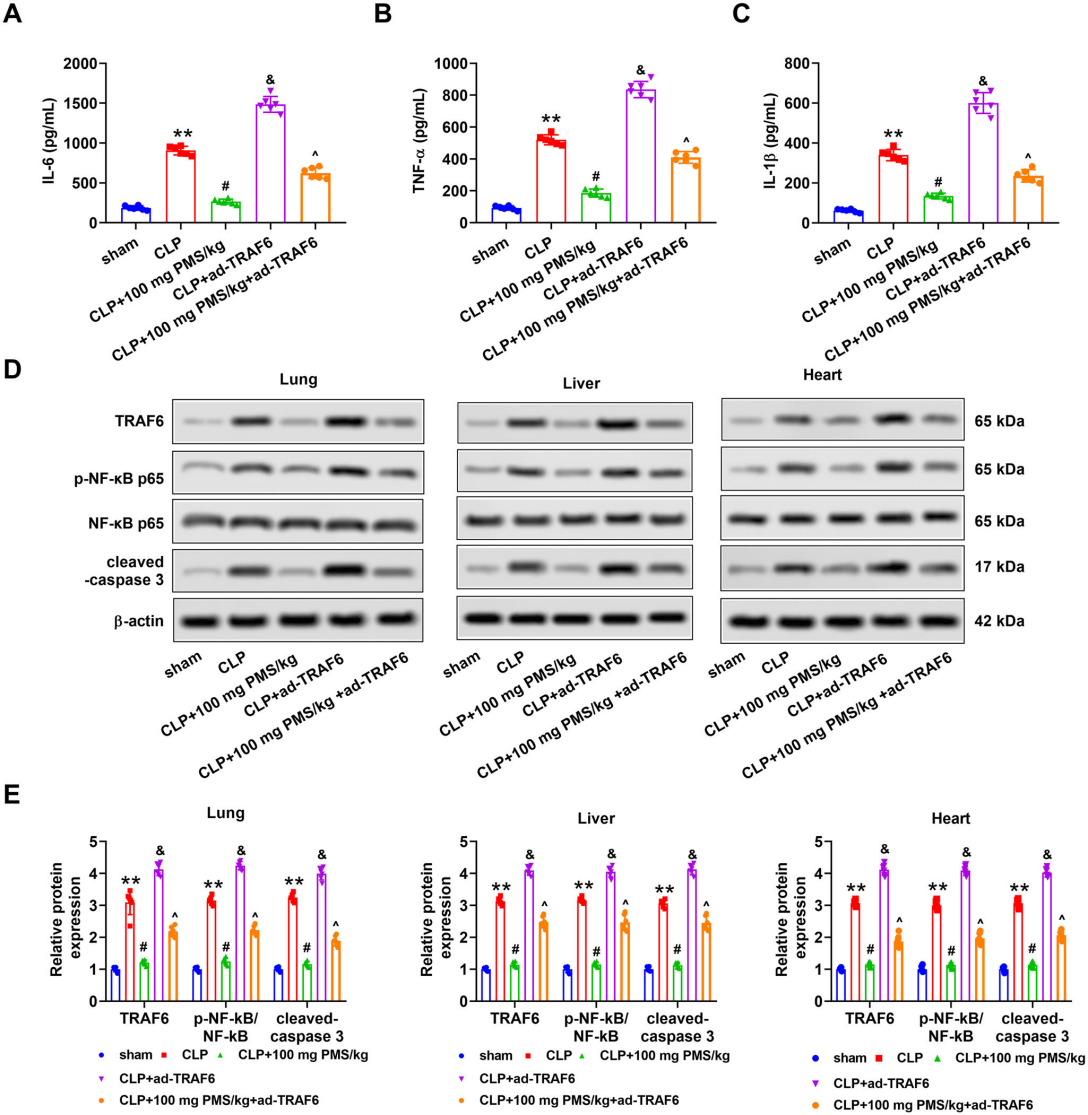
PMS remitted sepsis-triggered apoptosis and inflammation *via* mediating the TRAF6/NF-κB pathway. After transfection, the mice were divided into five groups, including sham, CLP, CLP + 100 mg PMS/kg, CLP + ad-TRAF6 and CLP + 100 mg PMS/kg + ad-TRAF6. (A–C) The contents of IL-6, TNF-α and IL-1β were analysed via ELISA. (D–E) Protein levels of TRAF6, p-NF-κB p65 and NF-κB and cleaved caspase 3 in lung, liver and heart tissues were determined by Western blotting. *p < 0.01, *vs.* Sham or ad-NC group; ^#,&^*p* < 0.05, *vs.* CLP group; ^^^*p* < 0.05, *vs.* CLP + ad-TRAF6 group.

The effects of the TRAF6/NF-κB pathway on PMS regulation of sepsis-induced apoptosis and inflammation were further explored. We noticed that overexpression of TRAF6 significantly enhanced the serum levels of IL-6, TNF-α and IL-1β from lung, liver and heart tissues in the CLP model (*p*** **<** **0.05). After pre-treatment with PMS (100 PMS mg/kg), the ascending changes of IL-6, TNF-α and IL-1β levels induced by ad-TRAF6 were restrained (*p*** **<** **0.05, Figure 6(A–C)). Furthermore, the protein levels of TRAF6, p-NF-κB p65 and cleaved caspase 3 in lung, liver and heart tissues were clearly heightened in the CLP + ad-TRAF6 group relative to that in the CLP group (*p*** **<** **0.05). Similar with above-mentioned results, the increased phenomenon triggered by TRAF6 overexpression was overturned by PMS (100 mg PMS/kg) treatment (*p*** **<** **0.05, Figure 6(D,E). The consequences indicated that the TRAF6/NF-κB pathway influenced the protective effect of PMS in sepsis-triggered apoptosis and inflammation.

## Discussion

Sepsis is a systemic inflammatory syndrome as a result of multifarious pathogenic microorganisms, which can trigger septic shock and even multiple organ failure (Torres et al. 2022). Currently, the mechanism of organ injury in sepsis is ambiguous, which might be linked to the imbalance of inflammatory response, activation of the NF-κB pathway and oxygen free radical scavenging disorder (Li J et al. 2017; Li Y-M et al. 2019). Numerous studies have confirmed that TCM has unique advantages and broad prospects in the prevention and treatment of sepsis and organ damage (Zhao et al. 2019; Fan et al. 2020). As a significant component of TCM, PMS has been applied to remedy various inflammation-related diseases (Son et al. 2017; Liu F et al. 2019). However, the role of PMS in sepsis-mediated inflammatory injury is still unknown. The present consequences indicated that PMS improved sepsis-triggered organ damage through regulating apoptosis and inflammatory response. This beneficial function of PMS in sepsis was mediated by the TRAF6/NF-κB pathway.

PMS is important component of Chinese herb *Plantago asiatica*, which has a wide range of curative function in multifold illnesses (Zan et al. 2019; Yu et al. 2022). The important evidence demonstrated that PMS mitigated lipopolysaccharide (LPS)-mediated pulmonary injury *via* inhibiting the NF-κB/MAPK axis (Wu H et al. 2016). Moreover, PMS exhibited the anti-fibrosis effect in liver through restraining the activation of hepatic stellate cells (Wang Y and Yan 2019). MPO and BLAF proteins are important indexes to evaluate the degree of lung tissue injury (Wang S et al. 2020). ALT and AST are transaminase reflecting the situation of liver injury, which are mainly indicators to detect liver function in clinic (Yip et al. 2021). CK is an important energy regulating enzyme directly related to intracellular energy transport, muscle contraction and ATP regeneration (Lima et al. 2021). The greatest value of CK and CK-MB in clinical application is the diagnosis of myocardial infarction (Wei et al. 2021). In this research, we explored the effect of PMS in sepsis-mediated organ damage through examining the above-mentioned indicators. The results indicated that PMS eased lung, liver and heart damage in the sepsis mouse model *via* prohibiting correlated factors.

Systemic inflammation is the main reason of sepsis death, which can develop towards respiratory distress syndrome (Papafilippou et al. 2020). Therefore, the inhibition of inflammation is of great significance in the treatment of organ damage caused by sepsis. The anti-inflammatory activity of PMS, as evidenced by reducing TNF-α, IL-1β and IL-6 levels in high glucose (HG)-triggered HBZY-1 cells, was uncovered (Xiao et al. 2021). Generally speaking, IL-1 initiates inflammatory response through expression of IL-1β, and IL-1β and IL-6 damage endothelial cells by activating granulocytes, releasing oxygen free radicals and metabolites thereby causing tissue damage (Li R et al. 2018). Moreover, studies have shown that inhibition of IL-6 expression could improve myocardial damage, while the synergistic action of TNF-α and IL-1β could cause myocardial damage in sepsis shock (Frencken et al. 2018; Wu B et al. 2020). Herein, we also discovered that PMS alleviated sepsis-triggered inflammation through decreased the levels of IL-6, TNF-α and IL-1β. Notably, crucial research disclosed that PMS induced cell apoptosis through modulating the expression levels of apoptosis-related genes (Bax, Bcl-2 and cleaved caspase 3) and activation of the PI3K/AKT signal pathway (Wang S et al. 2020). Consistent with the above research, we also found that PMS reduced sepsis-triggered apoptosis. This evidence uncovered the protective effect of PMS in sepsis-induced inflammation and apoptosis.

The NF-κB signalling pathway is one of the core pathways of the pathogenesis of sepsis, and the development of drugs to interfere with the NF-κB signalling pathway is considered to be a key method to reduce the mortality of sepsis (Cai et al. 2019). Tumour necrosis factor receptor-associated factor (TRAF) family is a genetically conserved connector protein involved in extensive biological functions, which plays a vital role in the immunologic process (Shi and Sun 2018). It has been demonstrated that TRAF6 is associated with sepsis-induced acute lung injury, and highly expressed TRAF6 could notably enhance the levels of TNF-α and IL-6 (Liu JH et al. 2021). Furthermore, one study demonstrated that dehydrocorydaline exerted the protective role against sepsis-mediated myocardial injury *via* regulating the TRAF6/NF-κB pathway (Li Y et al. 2021). Similarly, additional research has indicated that pellino1 aggravated the inflammatory response in sepsis-triggered lung injury through modulating the TRAF6/NF-κB pathway (Liu JH et al. 2021). In this research, we noticed that PMS treatment significantly reduced TRAF6 and p-NF-κB protein levels in the lung, liver and heart tissues of the sepsis mouse model. Interestingly, TRAF6 overexpression reversed the functions of PMS in sepsis-mediated organ damage, apoptosis and inflammation. These findings implied that the TRAF6/NF-κB pathway participated in regulating the effect of PMS in the progress of sepsis.

## Conclusions

Taken together, the present research indicated that PMS relieved sepsis-triggered organ dysfunction and inflammation, which was modulated by the TRAF6/NF-κB pathway. The research provided more ideas and directions for exploration the drugs with reliable clinical efficacy, and little toxic and side effects in the treatment of sepsis. Further experiments are still necessary for investigating the other regulated signals of PMS in sepsis-mediated injury.

## Data Availability

The data used and analysed during the current research are available from the corresponding author upon reasonable request.
